# Essential Oil of *Artemisia annua* L.: An Extraordinary Component with Numerous Antimicrobial Properties

**DOI:** 10.1155/2014/159819

**Published:** 2014-04-01

**Authors:** Anna Rita Bilia, Francesca Santomauro, Cristiana Sacco, Maria Camilla Bergonzi, Rosa Donato

**Affiliations:** ^1^Department of Chemistry, University of Florence, Via Ugo Schiff 6, 50019 Sesto Fiorentino, Florence, Italy; ^2^Department of Health Sciences, University of Florence, Viale G.B. Morgagni 48, 50134 Florence, Italy

## Abstract

*Artemisia annua* L. (Asteraceae) is native to China, now naturalised in many other countries, well known as the source of the unique sesquiterpene endoperoxide lactone artemisinin, and used in the treatment of the chloroquine-resistant and cerebral malaria. The essential oil is rich in mono- and sesquiterpenes and represents a by-product with medicinal properties. Besides significant variations in its percentage and composition have been reported (major constituents can be camphor (up to 48%), germacrene D (up to 18.9%), artemisia ketone (up to 68%), and 1,8 cineole (up to 51.5%)), the oil has been subjected to numerous studies supporting exciting antibacterial and antifungal activities. Both gram-positive bacteria (*Enterococcus*, *Streptococcus*, *Staphylococcus*, *Bacillus*, and *Listeria* spp.), and gram-negative bacteria (*Escherichia*, *Shigella*, *Salmonella*, *Haemophilus*, *Klebsiella*, and *Pseudomonas* spp.) and other microorganisms (*Candida*, *Saccharomyces*, and *Aspergillus* spp.) have been investigated. However, the experimental studies performed to date used different methods and diverse microorganisms; as a consequence, a comparative analysis on a quantitative basis is very difficult. The aim of this review is to sum up data on antimicrobial activity of *A. annua* essential oil and its major components to facilitate future approach of microbiological studies in this field.

## 1. Introduction

**Figure 1:**
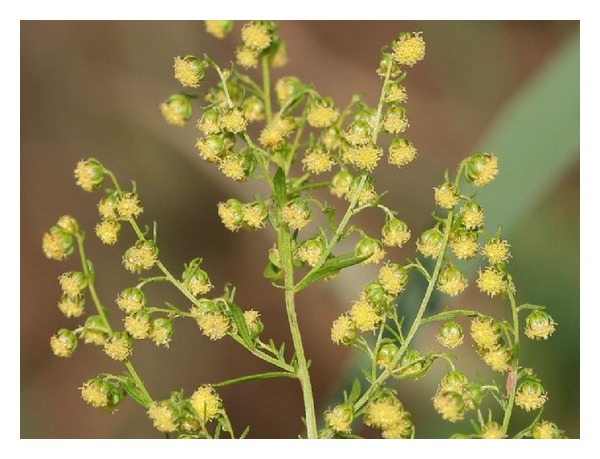
Picture of* A. annua* flowers (from http://upload.wikimedia.org/wikipedia/commons/5/59/Artemisia_annua_detail.jpeg).

*Artemisia annua* L., a plant belonging to the Asteraceae family, is an annual herb native to China and it grows naturally as a part of steppe vegetation in northern parts of Chatar and Suiyan province in China at 1,000–1,500 m above sea level. This plant can grow up to 2.4 m tall. The stem is cylindrical and branched. Leaves are alternate, dark green, or brownish green. Odour is characteristic and aromatic while the taste is bitter. It is characterized by large panicles of small globulous capitulums (2-3 mm diameter), with whitish involucres, and by pinnatisect leaves which disappear after the blooming period, characterised by small (1-2 mm) pale yellow flowers having a pleasant odour (Figure 1). The Chinese name of the plant is Qinghao (or Qing Hao or Ching-hao which means green herb). Other names are wormwood, Chinese wormwood, sweet wormwood, annual wormwood, annual sagewort, annual mugwort, and sweet sagewort. In the USA, it is well known as sweet Annie because after its introduction in the nineteenth century it was used as a preservative and flavouring and its aromatic wreath made a nice addition to potpourris and sachets for linens and the essential oil obtained from the flowering tops is used in the flavouring of vermouth [1]. The plant is now naturalised in many other countries such as Australia, Argentina, Brazil, Bulgaria, France, Hungary, Italy, Spain, Romania, the United States, and the former Yugoslavia [2].

Due to the presence of the unique sesquiterpene endoperoxide lactone artemisinin (Qinghaosu), one of the most important plant-derived drug in the treatment of the chloroquine-resistant and cerebral malarias, the plant is cropped on a large scale in China, Vietnam, Turkey, Iran, Afghanistan, and Australia. In India, it is cultivated on an experimental basis in the Himalayan regions, as well as temperate and subtropical conditions [3].

The essential oil which is rich in mono- and sesquiterpenes represents another source of potential commercial value [4]. Besides significant variations in its percentage and composition have been reported, it has been successfully subjected to numerous studies which mainly concern the antibacterial and antifungal activities. Diverse experimental studies have been reported to date using different methods and testing different microorganisms; therefore, a comparative analysis on a quantitative basis is very difficult. The aim of our review is to sum up data on antimicrobial activity of* A. annua* volatiles and its major components to facilitate future approach of microbiological experimental in this field.

## 2. Plant Distribution and Yield of the Volatiles

Essential (volatile) oil of* A. annua* can reach yields of 85 kg/ha. It is synthesised by secretory cells, especially of the uppermost foliar portion of the plant (top 1/3 of growth at maturity) which contains almost double number if compared with the lower leaves. It is reported that 35% of the mature leaf surface is covered with capitate glands which contain the terpenoidic volatile constituents. Essential oil from* A. annua* is distributed, with 36% of the total from the upper third of the foliage, 47% from the middle third, and 17% from the lower third, with only trace amounts in the main stem side shoots and roots. The yield of the oil generally ranges between 0.3 and 0.4% but it can reach 4.0% (V/W) from selected genotypes. Several studies have permitted the conclusion that* A. annua* crop could be harvested much before onset of flowering for obtaining high yields of artemisinin and the crop must be allowed to attain maturity to obtain high yields of the essential oil [5, 6].

Yield (herbage and essential oil content) can be increased with added nitrogen and the greatest growth was obtained with 67 kg N/ha. Increasing density of plants tended to increase essential oil production on an area basis, but the highest essential oil yields (85 kg oil/ha) were achieved by the intermediate density at 55,555 plants/ha receiving 67 kg N/ha. Finally the planting date and harvest time can influence the maximum concentration of the produced essential oil [6].

## 3. Chemical Profile of the Essential Oil

The essential oil, generally obtained by hydrodistillation of the flowering tops, analysed with GC-MS, revealed a great variability both in the qualitative and quantitative composition.

Chemical profile is generally influenced by the harvesting season, fertilizer and the pH of soils, the choice and stage of drying conditions, the geographic location, chemotype or subspecies, and choice of part plant or genotype or extraction method. In Table 1, the main constituents (>4%) of the investigated samples are reported.

Analysis of* A. annua* essential oils revealed the presence of mainly monoterpenoids and sesquiterpenes and the profiles showed great differences in the three main components, artemisia ketone, 1,8-cineole, and camphor, depending on the global phytogeographic origin. Oils can be grouped into the following:

- Vietnamese oil with 3.3–21.8% camphor and 0.3–18.9% germacrene D,
- Chinese oil with high content of artemisia ketone (64%),
- Indian oil with 11.5–58.8% of artemisia ketone,
- French oil with 2.8–55% artemisia ketone, 1.2–11.6% 1,8-cineole, and 15% germacrene D,
- North American oil with 35.7–68% artemisia ketone and 22.8–31.5% 1,8-cineole,
- Iranian oil with 48% camphor and 9.4% 1,8-cineole.

The presence of volatile oil is also reported in fruits and roots. Sesquiterpenes are the most abundant chemicals identified in the essential oil of the fruits; in particular, caryophyllene oxide (9.0%), caryophyllene (6.9%), (*E*)-*β*-farnesene (8.2%), and germacrene D (4.0%) are identified. However, only 52% of the total components were identified [7].

Upon hydrodistillation, the dried roots of* Artemisia annua* L. cultivar Jwarharti, a pleasantly fragrant essential oil, have been obtained with a yield of 0.25%. The oil was rich in sesquiterpenes and oxygenated sesquiterpenes and had* cis*-arteannuic alcohol (25.9%), (*E*)-*β*-farnesene (6.7%), *β*-maaliene (6.3%), *β*-caryophyllene (5.5%), caryophyllene oxide (4.4%), and 2-phenylbenzaldehyde (3.5%) as its major components [8].

Recently, the analysis of aromatic waters, obtained from plants collected at full blooming, showed the presence, among others, of camphor (27.7%), 1,8-cineole (14%), artemisia ketone (10.1%), *α*-terpineol (6.1%),* trans*-pinocarveol (5.4%), and artemisia alcohol (2%). From plants at the preflowering stage, aromatic waters gave camphor (30.7%), 1,8-cineole (12.8%), artemisia alcohol (11.4%), artemisia ketone (9.5%), alpha-terpineol (5.8%), and* trans*-pinocarveol (3.0%) as the main constituents. The qualitative and quantitative profiles of the two aromatic waters were similar [5].

## 4. Antimicrobial Activities of the Essential Oils

The essential oil of* Artemisia annua* has been the subject of numerous studies to test the antibacterial and antifungal activity. Tests were carried out both on the whole oil (Table 2) and on its principal components such as camphor, 1,8-cineol, *α*-pinene, and artemisia ketone (Table 3). The main gram-positive bacteria tested with* A. annua* volatiles obtained by hydrodistillation were* Staphylococcus aureus* [9–14],* Enterococcus hirae* [12],* Enterococcus faecalis* [14],* Streptococcus pneumoniae*,* Micrococcus luteus* [9],* Bacillus cereus* [14],* Sarcina lutea* [10],* Bacillus subtilis* [9, 11],* Bacillus thuringiensis* [11],* Bacillus* spp. [14], and* Listeria innocua* [15]. The gram-negative* Escherichia coli* [9, 11–14],* Escherichia coli* UPEC-Uropathogenic [14],* Escherichia coli* ETEC-Enterotoxigenic [16],* Escherichia coli* EPEC-Enteropathogenic [16],* Escherichia coli* EIEC-Enteroinvasive [16],* Escherichia coli* STEC-Shiga-toxin producer [16],* Shigella* sp. [10],* Salmonella enteritidis* [10],* Klebsiella pneumoniae* [10],* Haemophilus influenzae* [9], and* Pseudomonas aeruginosa* [9, 13, 14] were tested. Some strains of yeasts including* Candida albicans* [10, 12, 13],* Candida krusei* [9], and* Saccharomyces cerevisiae* [12, 13] and molds like* Aspergillus fumigatus* [10] were also tested (Table 2).

The main gram-positive bacteria tested with methanol, chloroform, ethanol, hexane, and petroleum ether extracts of* A. annua* were* Staphylococcus aureus* [14, 17],* Enterococcus faecalis* [14],* Micrococcus luteus* [17],* Bacillus cereus* [14, 17],* Bacillus subtilis* [17],* Bacillus pumilus* [17], and* Bacillus* sp. [14]. The gram-negative* Escherichia coli* [14, 17],* Escherichia coli* UPEC [14],* Salmonella typhi* [14, 17], and* Pseudomonas aeruginosa* [14, 17] were tested.

In addition, several single main components were investigated (Table 3), including *α*-terpineol [18] tested on* C. albicans*,* C. glabrata*,* C. dubliniensis*,* C. guilliermondii*,* C. krusei*,* C. parapsilosis*, and* C. tropicalis*; artemisia ketone, *α*-pinene, 1,8-cineole, and camphor [10] tested on* C. albicans*,* B. cereus*,* S. aureus*,* S. lutea*,* E. coli*,* K. pneumoniae*,* Ps. aeruginosa*,* S. enteritidis*,* Shigella* sp., and* A. fumigatus.*

The antifungal activity of the essential oil was also evaluated against economically important foliar and soil-borne fungal pathogens of tomato. The essential oil was active against* Sclerotinia sclerotiorum*,* Botrytis cinerea*,* Phytophthora infestans*, and* Verticillim dahliae* [19].

Different methods were used to evaluate the antibacterial and antifungal properties and included agar disk diffusion method [9, 11, 14, 17], minimal inhibition concentration (MIC) [9, 10, 12–14, 16–18], minimal bacterial concentration (MBC) [10, 14], and minimal fungicidal concentration (MFC) [10, 18] as reported in Table 2.

The results related to agar disk diffusion method (Table 2) show that some important pathogens are sensitive to* A. annua* essential oil obtained by hydrodistillation.* S. aureus*,* S. pneumoniae*,* E. coli*, UPEC,* H. influenzae*,* P. aeruginosa*,* C. albicans*, and* C. krusei* were inhibited by the action of the oil.* H. influenzae*,* S. pneumoniae*, and* C. krusei* were more sensitive; their inhibition zones diameters were >60, 50, and 30 mm, respectively. Satisfactory results were also achieved with genus* Bacillus*. On the contrary,* M. luteus* and* L. innocua* were resistant to this essential oil. Since the use of agar disk diffusion method is limited by the hydrophobic nature of most essential oils and plant extracts components that prevents their uniform diffusion through the agar medium, the most authors report the results obtained with MIC and MBC methods.

However, from the literature it is observed that the results obtained by agar disk diffusion method were confirmed by the liquid medium methods (MBC and MIC). At present there is no complete agreement on the concentration of the extracts to be considered active or inactive. Duarte and coworkers [16] proposed a classification to be applied to the extracts based on MIC values; this author considers MIC up to 500 *μ*g/mL as strong inhibitors, MIC between 600 and 1500 *μ*g/mL as moderate inhibitors, and MIC above 1600 *μ*g/mL as weak inhibitors. In recent years, many different microbial species of medical interest have been tested from which emerged encouraging results except in the case of* E. coli* with special pathogenic characters (ETEC, EPEC, EIEC, and STEC) sensitive only at high concentrations of the extracts.

As concerns the results obtained against fungal strains, the data are rather limited. The results are contrasted against* C. albicans* but have to be more explored, while data related to* A. fumigatus* and* C. krusei* are encouraging.

Further studies have been performed with the main components present in* A. annua* essential oil (see Table 3). These studies show that artemisia ketone is the component of the oil that has the greatest antimicrobial activity; in fact, it always turns out to be effective against bacteria and some fungi (*C. albicans* and* A. fumigatus*) at very low concentrations (range 0.07–10 mg/mL). The other compounds tested in the studies have produced variable results; however, it should be emphasized the fact that all the compounds tested by liquid methods were active (range 1.25–5 mg/mL) against* A. fumigatus*, a dangerous microorganism frequently responsible for nosocomial infections in immunosuppressed subjects.

## 5. Concluding Remarks

During the last decade several authors have evaluated the antimicrobial activity of* Artemisia annua* and some of its main components. The composition of the essential oil shows great differences in the three main characteristic components, namely, artemisia ketone, 1,8-cineole, and camphor, depending on the global phytogeographic origin. Besides the different chemical profiles, artemisia essential oil has revealed strong antimicrobial properties towards numerous bacterial strains, both gram-positive and gram-negative, and diverse fungal strains, including many pathogens. Biological effects are the result of a synergism of all molecules contained in an essential oil, even if it is possible that the activity of the main components is modulated by other minor molecules, but the activity of the isolated constituents is also remarkable.* Artemisia annua* volatile constituents appear to be a resource of many biologically active compounds which will hopefully give new economically important by-product. The good results obtained encourage further researches aiming at a possible application of these substances in food and pharmaceutical and cosmetology fields.

## Figures and Tables

**Table 1:** Compounds (>4%) isolated from essential oil of *Artemisia annua* L.

Compound	Country	%	Reference
Artemisia alcohol	China (Cult)	7.5	[20]
	USA-CA	5.2	[21]
	Serbia	4.8	[10]

Artemisia ketone	Not stated	38.0	[22]
	France	52.5	[23]
	Serbia	35.7	[10]
	Egypt	13.9	[15]
	China	2.21	[11]
	Bosnia	30.7	[9]
	USA-CA	35.7	[24]
	China (Cult)	63.9	[20]
	USA-IN	68.5	[25]
	England	61.0	[26]
	Vietnam	0.1–4.4	[27]
	Indian (Cult)	58.8	[28]
	India (Cult)	11.5	[29]
	Turkey	22	[19]

Borneol	Not stated	20.0	[22]
	England	7.0	[26]
	China (Cult)	15.9	[30]

Camphene	Iran	7	[13]

Camphene hydrate	USA-IN	12.0	[25]

Camphor	Vietnam	21.8	[3]
	Serbia	4.2	[10]
	Egypt	5.08	[15]
	France	27.5	[23]
	China (Cult)	21.8	[20]
	Vietnam (Cult)	3.3	[20]
	Bosnia	15.8	[9]
	Iran	1.92	[14]
	Italy	17.6	[5]
	Indian (Cult)	15.75	[28]
	India (Cult)	8.4	[29]
	France	43.5	[12]
	Iran	48	[13]
	Turkey	31	[19]

*Trans*-Cariophyllene	Egypt	7.73	[15]

*β*-Caryo phyllene	Italy	9.0	[5]
	Vietnam (Cult)	5.6	[20]
	Vietnam	3.3–8.6	[27]
	China (Cult)	5.98	[30]
	India (Cult)	12.2	[29]
	France	8.9	[12]

Caryophyllene oxide	China	5.13	[11]

Chrysanthenone	Vietnam	1.1–7.3	[27]
	India (Cult)	10.19	[28]

1,8-Cineol	France	11.66	[23]
	Serbia	5.5	[10]
	Egypt	8.13	[15]
	Bosnia	4.8	[9]
	USA-IN	22.8	[25]
	USA-CA	31.5	[21]
	Iran	9.4	[13]
	Iran	11.4	[14]
	Turkey	10	[19]

*β*-Farnesene	Italy	10.2	[5]
	Vietnam	1.1–12.8	[27]
	Egypt	5.32	[15]
	China (Cult)	12.9	[30]

Germacrene D	Vietnam (Cult)	18.3	[20]
	Italy	21.2	[5]
	Vietnam	0.3–18.9	[27]
	China (Cult)	10.9	[30]
	France	15.6	[12]

*α*-Guaiene	China (Cult)	4.7	[20]

Linalool	Vietnam	0.1–4.2	[27]
	Iran	8.1	[14]

Linalool acetate	England	10.0	[22]

Myrcene	China (Cult)	5.1	[20]
	USA-CA	4.6	[21]
	Vietnam	0.1–8.5	[27]

*α*-Pinene	USA-CA	11.2	[21]
	USA-IN	16.0	[25]
	Serbia	16.5	[10]

(*Trans*)-Pinocarveol	France	10.9	[12]
	Serbia	4.8	[10]

Sabinene	France	9.4	[12]

Spathulenol	Iran	4.97	[14]
	Iran	4.9	[13]

**Table 2:** Tests carried out on the whole oil.

Bacterial strains	Agar disk diffusion	Concentration	Reference	MIC	mg/mL	Reference	MBC	mg/mL	Reference
Gram-positive									
*S. aureus *	Not active		[12]						
				Active	32	[13]			
	Active	5.00%	[11]	Active	0.0156–0.0313	[11]			
	Active	10 mg/mL	[9]						
				Active	5.0–10.0	[10]	Low activity	>20.0–10.0	[10]
	Active	10 mg/mL	[14]	Active	0.031	[14]	Active	0.031	[14]
*E. hirae *	Active	0.1 mg/mL	[12]						
*E. faecalis *	Active		[9]						
	Active	10 mg/mL	[14]	Active	0.026	[14]	Active	0.031	[14]
*S. pneumoniae *	Active	10 mg/mL	[9]						
*M. luteus *	Not active		[9]						
*B. cereus *	Active	10 mg/mL	[14]	Active	0.053	[14]	Active	0.055	[14]
				Low activity	20	[10]	Low activity	20	[10]
*B. subtilis *	Active	5.00%	[11]	Active	0.00781-0.00781	[11]			
	Active	10 mg/mL	[9]						
*B. thuringensis *	Active	5.00%	[11]	Active	0.0313–0.0156	[11]			
*B. *sp.	Active	10 mg/mL	[14]	Active	0.026	[14]	Active	0.053	[14]
*L. innocua *	Not active		[15]						
*Sarcina lutea *				Active	2.5	[10]	Active	2.5	[10]
Gram-negative									
*E. coli *	Not active		[12]						
				Active	64	[13]			
	Active	5.00%	[11]	Active	0.0313-0.0313	[11]			
	Active	10 mg/mL	[9]						
				Low activity	20	[10]	Low activity	20	[10]
	Active	5 mg/mL	[14]	Active	0.017	[14]	Active	0.024	[14]
UPEC	Active	5 mg/mL	[14]	Active	0.026	[14]	Active	0.031	[14]
*Shigella *sp.				Low activity	20	[10]	Low activity	20	[10]
*S. enteritidis *				Active	5	[10]	Low activity	20	[10]
*K. pneumoniae *				Low activity	20	[10]	Low activity	20	[10]
*H. influenzae *	Active	10 mg/mL	[9]						
*P. aeruginosa *				Not active		[13]			
	Active	10 mg/mL	[9]						
	Active	10 mg/mL	[14]	Active	0.025	[14]	Active	0.053	[14]

Fungal strains	Agar disk diffusion	Concentration	Reference	MIC	mg/mL	Reference	MFC	mg/mL	Reference

*C. albicans *	Active	0.2 mg/mL	[12]						
				Active	2	[13]			
				Low activity	20	[10]	Not active	>20	[10]
*C. krusei *	Active	10 mg/mL	[9]						
									
*S. cerevisiae *	Active	0.2 mg/mL	[12]						
				Active	2	[13]			
*A. fumigatus *				Active	5	[10]	Active	5	[10]

**Table 3:** Tests on the main components of *A. annua* essential oil.

	Artemisia ketone		*α*-Pinene		1,8-Cineole		Camphor		Reference	*α*-Terpineol		Reference
	MIC (mg/mL)	MBC (mg/mL)	MIC (mg/mL)	MBC (mg/mL)	MIC (mg/mL)	MBC (mg/mL)	MIC (mg/mL)	MBC (mg/mL)		MIC (%, v/v)	MBC (%, v/v)	
Bacterial strains												
Gram- positive												
*S. aureus *	0.07–0.15	0.3–0.6	>10	>10	2.5–5	2.5–5	2.5–5	2.5–>10	[10]			
*Sarcina lutea *	2.5	10	1.25	2.5	0.6	1.25	2.5	2.5	[10]			
*B. cereus *	0.6	0.6	>10	>10	20	20	10	10	[10]			
Gram- negative												
*E. coli *	10	10	>10	>10	20	20	>10	>10	[10]			
*Shigella *sp.	0.6	0.6	>10	>10	10	20	>10	>10	[10]			
*S. enteritidis *	0.6	10	0.6	5	5	10	>10	>10	[10]			
*K. pneumoniae *	2.5	2.5	>10	>10	5	5	1.25	5	[10]			
Fungal strains												
*C. albicans *	10	10	>10	>10	5	20	5	5	[10]	0.25	0.5	[18]
*C. glabrata *										0.12	0.5	[18]
*C. dubliniensis *										0.12	0.25	[18]
*C. krusei *										0.12	0.5	[18]
*C. guillermondii *										0.12	0.25	[18]
*C. parapsilosis *										0.06	0.5	[18]
*C. tropicalis *										0.5	0.5	[18]
*A. fumigatus *	2.5	2.5	5	5	1.25	2.5	2.5	2.5	[10]			
